# The ‘Common Disease-Common Variant’ Hypothesis and Familial Risks

**DOI:** 10.1371/journal.pone.0002504

**Published:** 2008-06-18

**Authors:** Kari Hemminki, Asta Försti, Justo Lorenzo Bermejo

**Affiliations:** 1 Division of Molecular Genetic Epidemiology, German Cancer Research Center (DKFZ), Heidelberg, Germany; 2 Center for Family and Community Medicine, Karolinska Institute, Huddinge, Sweden; Erasmus University Medical Center, Netherlands

## Abstract

The recent large genotyping studies have identified a new repertoire of disease susceptibility loci of unknown function, characterized by high allele frequencies and low relative risks, lending support to the common disease-common variant (CDCV) hypothesis. The variants explain a much larger proportion of the disease etiology, measured by the population attributable fraction, than of the familial risk. We show here that if the identified polymorphisms were markers of rarer functional alleles they would explain a much larger proportion of the familial risk. For example, in a plausible scenario where the marker is 10 times more common than the causative allele, the excess familial risk of the causative allele is over 10 times higher than that of the marker allele. However, the population attributable fractions of the two alleles are equal. The penetrance mode of the causative locus may be very difficult to deduce from the apparent penetrance mode of the marker locus.

## Introduction

The common disease-common variant (CDCV) hypothesis posits that common, interacting disease alleles underlie most common diseases, perhaps in association with environmental factors [1], [2]. This hypothesis has been the scientific paradigm for genome-wide association (GWA) studies that have been or are being conducted on many common diseases. Numbers of new susceptibility loci are being identified. For example, the recent study by the Wellcome Trust Case Control Consortium detected 24 independent association signals for 7 major diseases [3]. For prostate cancer, many independent susceptibility loci have been described, one of which also predisposes to colorectal cancer [4]–[11]. Typically, the detected variants are common, with a frequency (p) higher than 10%, they are associated with low genotype relative risk (GRR), they explain a large proportion of the disease occurrence (described by the population attributable fraction, PAF), they explain a tiny fraction of the familial risks (quantified by e.g., the sibling relative risk λs) and, notably, they are located in non-coding regions and the function of most identified variants is unknown [5], [7], [12], [13]. The search for functionality at many of the verified loci, such as 8q24 in prostate cancer, has revealed no clues to the mechanism of action [4]–[6]. The disparity between the high PAFs explained by the identified loci, approaching 100% for some diseases, and the low λs attributable to the detected associations has been noticed before [2], [3], [14]. For example for breast cancer, the joint PAF of the identified genes/loci is over 60% but they explain less than 30% of the familial aggregation [15]–[17]; for prostate cancer, no more than 15% of familial risk is explained [11], although the joint PAF is probably 100% considering the independent 8q24 signals and the large numbers of loci reported in the March 2008 issue of Nature Genetics [5], [6], [9]–[11]. These discrepancies appear [18] to challenge the CDCV paradigm, because the genes with a large population impact, PAF, also eventually need to explain the familial aggregation of the disease [14].

We test here a hypothesis that may help to understand the paradox of high PAFs and low λs. When the identified marker polymorphism is linked to a functional locus, the PAF for the functional ‘causative’ allele is equal to the PAF for the marker, but the familial risk attributable to the causative allele increases in concert with the rarity of the variant and its increasing GRR. In order to test the hypothesis, we model genetic parameters in terms of a marker and a causative allele and translate these into PAFs and λs [19].

## Results

**Figure 1:**
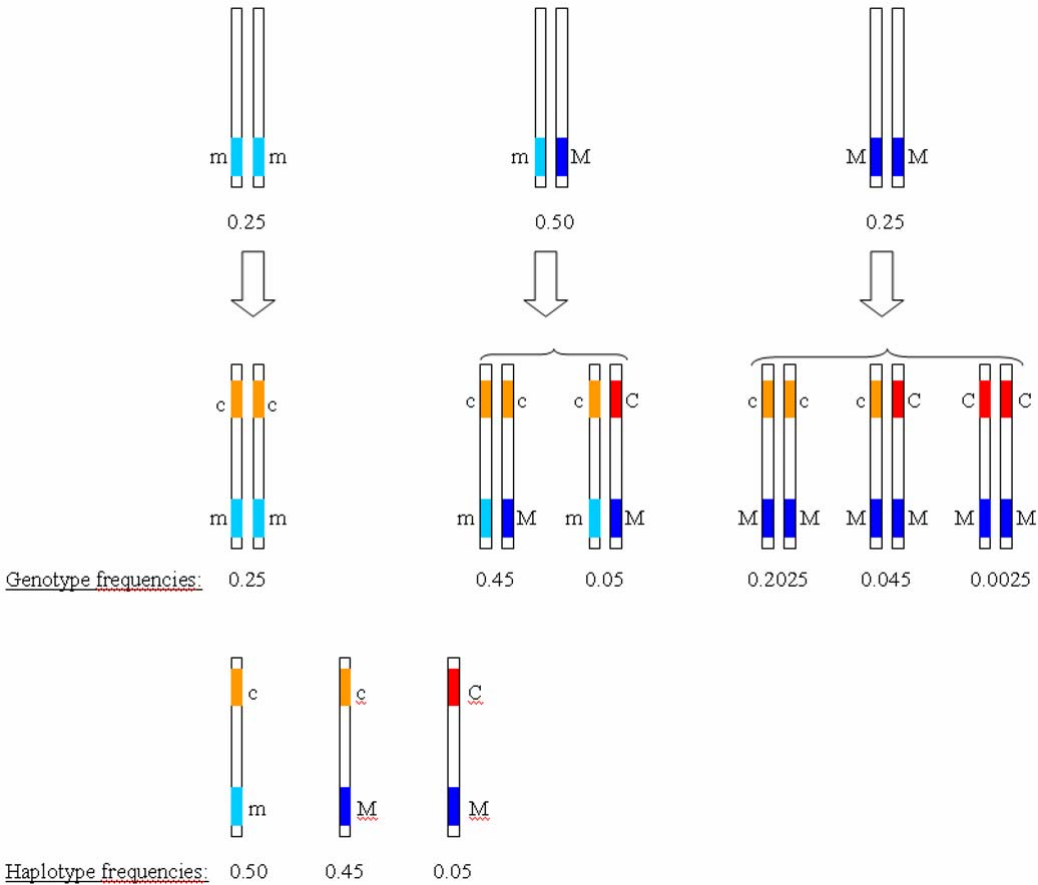
Use of a marker allele M to identify a causative locus C in genetic association studies. The frequency of M is pM = 0.5; the frequency of C is pC = 0.05; the coverage of C by M is complete (with D′ = 1.0). The genotype and haplotype frequencies are shown for the assumed parameters.

Fig. 1 shows a scheme on gene identification based on linkage disequilibrium. It is assumed that the marker allele M tags the causative variant C so that M is more frequent than C but C is always found together with M, i.e., D′ = 1.0. There are thus three haplotypes, c-m, c-M and C-M. The association signal for M is entirely due to the functional effect of C. The example shown in Table 1 assumes that M is common and that the frequency of C is 1/10 of that of M (p_M_ = 0.5, p_C_ = 0.05). We further assume dominant penetrance for C (GRR_C_Hom_ = GRR_C_Het_) and D′ = 1.0. When the true GRR of the causative allele C is 1.5, the GRR of M is 1.10 for homozygotes and 1.05 for heterozygotes. The explained familial risks would be 1.01 for C and 1.00 for M. Notably, PAF is 4.6% and it is equal for C and M. If the GRR for C equals 10, the GRRs for M are 2.71 for homozygotes and 1.90 for heterozygotes. The familial risks would be 2.00 for C and 1.05 for M. The PAF is 46.7%. These data show that the observed GRRs for M are essentially lower than the true GRRs for the causative allele. Moreover, a dominant causative allele may result in non-dominant associations between the marker and the disease. In fact, the penetrance mode of M in Table 1 is close to additive.

**Table 1:** Genotype relative risk (GRR), familial risk (sibling relative risk λs) and population attributable fraction (PAF) related to a marker M in linkage disequilibrium with a causative allele C.

Genotype relative risk (GRR)			Familial risk (λs)		PAF (%)
Causative allele C	Marker allele M		Causative allele C	Marker allele M	
GRR_C_Hom_ = GRR_C_Het_	GRR_M_Hom_	GRR_M_Het_	λs	λs	
1	1	1	1	1	0
1.5	1.10	1.05	1.01	1.00	4.6
2	1.19	1.10	1.04	1.00	8.9
5	1.76	1.40	1.36	1.02	28.1
10	2.71	1.90	2.00	1.05	46.7
20	4.61	2.90	2.93	1.10	64.9
50	10.31	5.90	4.12	1.16	82.7
100	19.81	10.9	4.75	1.20	90.6

The assumed parameters are p_M_ = 0.5, p_C_ = 0.05, dominant penetrance for the causative allele (GRR_C_Hom_ = GRR_C_Het_) and D′ = 1.0.

**Figure 2:**
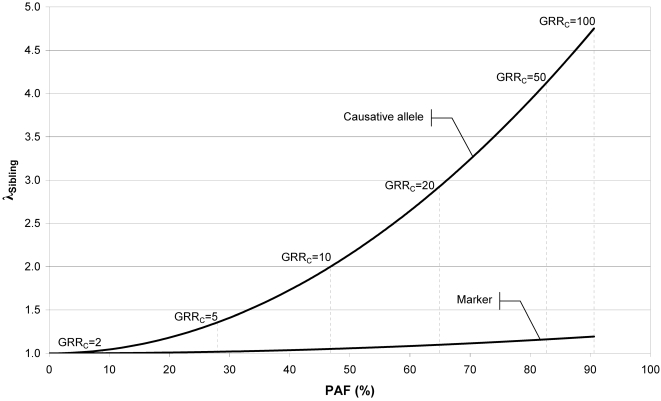
Relationship between the population attributable fraction (PAF) and the sibling relative risk (λs) for a causative locus C and marker allele M. The assumed parameters were pM = 0.5, pC = 0.05, dominant inheritance for C and D′ = 1.0.

Fig. 2 shows the relationship between the PAF and λs attributable to a causative allele and to a linked marker SNP. The obvious message from the graph is that, when D′ = 1.0, the PAFs explained by the causative allele and by the marker are equal. The relationship between λs and PAF is non-linear, the relative difference between GRR and λs for C over M increasing towards higher PAFs. The dependence of the relationship between PAF and λs on the frequency of the marker, the frequency of the causative allele, the inheritance mode of the causative allele and the extent of linkage disequilibrium is presented in the supporting information. In the supplementary figures, the top panels reproduces always Fig. 1, while the bottom panels show the effect of changing one parameter value at a time. When the frequency difference between M and C decreases to less that 1/10, the relative difference in their λs decreases (Fig. S1 and Fig. S2). When the penetrance mode of C is recessive, the relative difference between λs for C and M becomes very large (Fig. S3). When the linkage between M and C is incomplete (D′ = 0.9) the explained PAF by M and C differ (Fig. S4).

## Discussion

The first-generation WGA studies have been very successful and by March 2008 it has been estimated that some 100 loci have been associated with an increased risk of complex diseases [20]. However, in the midst of the jubilee, challenging questions are emerging. First, as many newly discovered loci lack a demonstrated function, the underlying mechanisms remain to be established. Second, the reported GRRs are small and they tend to decrease in the verification analysis, probably because in the relatively small initial WGA study chance contributed to the GRRs of the SNP selected for the verification, a phenomenon called ‘winner's curse’ or ‘the Beavis effect’ [11], [21]. When the GRRs are well below 1.5 there is a possibility of bias through an unmeasured environmental factor, as discussed in the context of nicotinic acetylcholine receptor and lung cancer risk [20]. Third, the results have shown the apparent discrepancy between the high PAF and the low λs, as discussed in the Introduction. Any positive results from the current WGA studies will have PAFs in excess of 5–10% because the WGA platforms contain HapMap described SNPs of high allele frequency (>5%) [19]. Even the ‘classical’ high-risk disease susceptibility genes explain a minor proportion of the observed familial aggregation for most diseases [3], [18]. For example, the high penetrant breast cancer genes, including BRCA1/2, are thought to explain less than 25% of the familial risk [12]. Similarly, the Wellcome Trust Case Control Consortium concludes that “the association signals so far identified account for only a small proportion of overall familiality” [3].

The present findings may help to interpret and use the results from GWA studies relating to the familial risk. Some association signals from loci of unknown function are likely to be markers of rarer causative variants which contribute significantly to the familial aggregation of the particular disease. Importantly, the λs of the causative and the marker loci are variable but their conferred PAFs remain identical if D′∼1.0. Thus the low familial risks for many of the replicated loci probably signal that they are markers of yet unidentified causative alleles.

Some recent studies support our hypothesis. For example, the NOD2 gene, which was the first identified susceptibility gene for Crohn disease [22], carries three susceptibility variants which account for most of the observed effects [23]. Two of the three variants are covered by the marker SNP (rs17221417) with D′ = 1.0. In the study of the Wellcome Trust Case Control Consortium, the GRR for Crohn disease was 1.92 for homozygote and 1.29 for heterozygote carriers [3]. The allele frequency of the SNP was 0.287, which results in λs = 1.02. By contrast, the familial risk attributable to variants in the NOD2 locus has been estimated to range from 1.19 to 1.49, depending on the population prevalence of the mutant alleles [23], [24]. This example illustrates the large difference in familial risks explained by markers and causative variants. It also shows that a single SNP, even a tagging SNP, may not capture all the genetic effects of the gene, thus causing an underestimation of the related familial risk.

Another important point from the present calculations is that the penetrance mode of the causative variant is very difficult to deduce from the apparent penetrance mode of the marker locus. Many of the published WGA studies discuss the apparent penetrance mode of the discovered loci, which is not warranted based on our results.

The WGA studies use linkage equilibrium between the marker and the causative locus as a mapping concept. The present results are a direct consequence of the mapping concept. The low λs explained by variants detected in recent genome scans may simply be due to their association with relatively rare causative variants. Moreover, the apparent penetrance modes of the marker genotypes may be misleading about those of the causative genotypes.

## Methods

Let p_C_ represent the frequency of a causative allele C in linkage disequilibrium with a marker SNP M. If the frequency of the marker is denoted by p_M_, the distribution of the four possible haplotypes is:
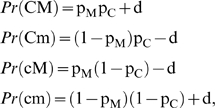
where d = D′(1−p_M_) p_C_, D′ being Lewontin's measure of linkage disequilibrium (see Figure 1 in the main text illustrating the concept of association between two alleles).

We assume that, given the genotype at the causal locus, the risk of disease is conditionally independent of the marker genotype. For example, if κ_0_ is the disease prevalence among individuals with wild type genotypes (G = cM/cM), the probability that an individual with genotype cM/cM is affected by the disease (A = 1) is given by *Pr*(A = 1∥G = cM/cM) = κ_0_, and
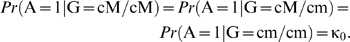
The relative risk of disease for homozygote carriers of C compared to wild type genotypes is:
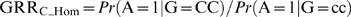
and the relative risk for heterozygotes compared to wild types is:
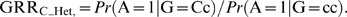
Then, the probability that an individual has the genotype CM/CM and he/she is affected is given by:
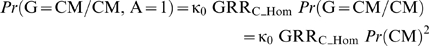
Similarly,
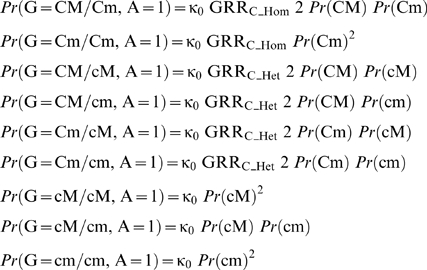
The previous equations can be used to calculate the disease prevalence according to the marker genotype:
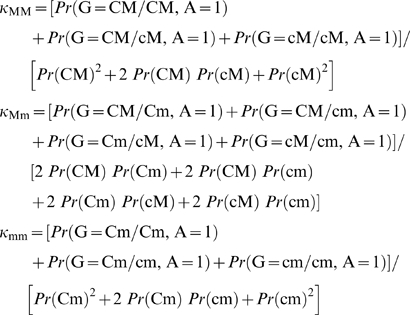
The genotype relative risks attributable to the marker are:
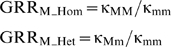



Next section describes the calculation of the population fraction (PAF) and the familial risk (sibling recurrence risk, λs) attributable to a causative allele C with frequency p_C_ and genotype relative risks GRR_C_Hom_ and GRR_C_Het_. The substitution in the formulas of the genetic parameters for the marker allele (p_M_, GRR_M_Hom_ and GRR_M_Het_) results in the corresponding estimates for M. The probability that an individual in the population is wild type homozygote (G = cc) and he is affected (A = 1) is:
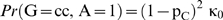
Similarly,
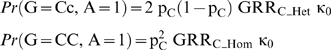



The prevalence of the disease in the population is then:
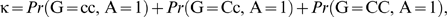
and the PAF is:
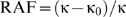



The sibling recurrence risk is given by:
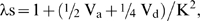
where V_a_ is the additive genetic variance divided by κ_0_ *^2^*, V_d_ is the dominance genetic variance divided by κ_0_ *^2^* and K = κ/κ_0_. V_a_ equals 2p_C_(1−p_C_)[(1−p_C_)(1−GRR_C_Het_)+p_C_(GRR_C_Het_−GRR_C_Hom_)]^2^ and V_d_ equals p_C_^2^(1−p_C_)^2^[1+GRR_C_Hom_−2GRR_C_Het_]^2^. Note that both the PAF and the λs are independent of the baseline prevalence κ_0_. Since κ = κ_0_/(1−PAF), the sibling risk can be also calculated as:
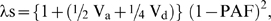

Supporting Information S1 provides the code for the above calculations.

## Supporting Information

Figure S1 Dependence of the relationship between population attributable fraction (PAF) and familial risk (λs) for a causative allele C in linkage disequilibrium with a marker M on the frequency of the marker pM. The assumed parameters are: frequency of the marker allele pM = 0.5 or pM = 0.1, frequency of the causative allele pC = 0.05, dominant inheritance of the causative allele (homozygous and heterozygous carriers of C are at similar risks of disease) and D' = 1.0.
(0.03 MB TIF)

Figure S2 Dependence of the relationship between population attributable fraction (PAF) and familial risk (λs) for a causative allele C in linkage disequilibrium with a marker M on the frequency of the causative allele pC. The assumed parameters are: frequency of the marker allele pM = 0.5, frequency of the causative allele pC = 0.05 or pC = 0.1, dominant inheritance of the causative allele (homozygous and heterozygous carriers of C are at similar risks of disease) and D' = 1.0.
(0.03 MB TIF)

Figure S3 Dependence of the relationship between population attributable fraction (PAF) and familial risk (λs) for a causative allele C in linkage disequilibrium with a marker M on the mode of inheritance. The assumed parameters are: frequency of the marker allele pM = 0.5, frequency of the causative allele pC = 0.05, dominant or recessive inheritance and D' = 1. 0. Note the different scaling of the two λs-axes.
(0.03 MB TIF)

Figure S4 Dependence of the relationship between population attributable fraction (PAF) and familial risk (λs) for a causative allele C in linkage disequilibrium with a marker M on the linkage disequilibrium. The assumed parameters are: frequency of the marker allele pM = 0.5, frequency of the causative allele pC = 0.05, dominant inheritance of the causative allele and D' = 1.0 or D' = 0.9.
(0.03 MB TIF)

Supporting Information S1 Code for calculation of PAFs and λs using the free software environment R (www.r-project.org)
(0.04 MB DOC)
