# Supplementary material for: The ‘Common Disease-Common Variant’ Hypothesis and Familial Risks
Source: PLoS One. 2008 Jun 18;3(6):e2504. doi: 10.1371/journal.pone.0002504 (PMC2423486; doi:10.1371/journal.pone.0002504)
Supplement: Supporting Information S1 — Code for calculation of PAFs and λs using the free software environment R (www.r-project.org) (0.04 MB DOC) [file pone.0002504.s005.doc]

Usage:

PAF_FRR_Power(GRR,pA,inher,k,ncases,ncontrols,iter)

Arguments:

GRR: the genotype relative risk

pA: the frequency of the susceptibility allele

inher: the inheritance mode (1 dominant, 2 recessive, 3 additive, 4

multiplicative)

k: the disease prevalence

ncases: number of cases

ncontrols: number of controls

iter: number of iterations. If iter=1, the power is not estimated

(POWER=999)

Value:

‘PAF_FRR_Power’ returns a vector with the components PAF, FRR and POWER

Author:

Justo Lorenzo Bermejo, Department of Molecular Genetic Epidemiology,

German Cancer Research Centre, Heidelberg, Germany

Examples:

# PAF and FRR attributable to BRCA1

PAF_FRR_Power(10,0.001,1,0.05,500,500,1)

# PAF, FRR and power for DG8S737 – 500 cases and 500 controls

PAF_FRR_Power(3.1329,0.078,4,0.05,500,500,1000)

Code:

## Calculation of PAF, FRR and power

PAF_FRR_Power<-function(GRR,pA,inher,k,ncases,ncontrols,iter) {

#PAFs and expected genotype distributions among cases/controls

f=0.0001;

enda=0;

if (inher==1) {GRR1=GRR; GRR2=GRR};

if (inher==2) {GRR1=GRR; GRR2=1};

if (inher==3) {GRR1=GRR; GRR2=(1+GRR)/2};

if (inher==4) {GRR1=GRR; GRR2=GRR**0.5};

while (enda==0){

f=f+0.00001;

#Genotypes among cases

uno1=(1-pA)*(1-pA)*f;

uno2=2*pA*(1-pA)*f*GRR2;

uno3=pA*pA*f*GRR1;

den=uno1+uno2+uno3;

kp=den;

pAA_case=uno1/den;

pAB_case=uno2/den;

pBB_case=uno3/den;

#Genotypes among controls

dos1=(1-pA)*(1-pA)*(1-f);

dos2=2*pA*(1-pA)*(1-f*GRR2);

dos3=pA*pA*(1-f*GRR1);

den=dos1+dos2+dos3;

pAA_cont=dos1/den;

pAB_cont=dos2/den;

pBB_cont=dos3/den;

PAF=100*(kp-f)/kp;

if (k <= kp) {enda=1};

}

#FRR

uno=pA*pA*GRR1;

dos=2*pA*(1-pA)*GRR2;

tres=(1-pA)*(1-pA);

denom=uno+dos+tres;

#Additive genetic variance divided by f

vauno=2*pA*(1-pA);

vados=(1-pA)*(1-GRR2);

vatres=pA*(GRR2-GRR1);

vacua=(vados+vatres)*(vados+vatres);

va=vauno*vacua;

#Dominance variance divided by f

vduno=pA*pA*(1-pA)*(1-pA);

vddos=1+GRR1-(2*GRR2);

vdtres=vddos*vddos;

vd=vduno*vdtres;

#SIBLING FRR

arriba=0.5*va+0.25*vd;

abajo=denom*denom;

FRR=1+(arriba/abajo);

if (iter>1){

#Power of a case-control study

one=pAA_case;

two=one+pAB_case;

thr=two+pBB_case;

onea=pAA_cont;

twoa=onea+pAB_cont;

thra=twoa+pBB_cont;

acaba=0;

rep=0;

sign=0;

while (acaba==0){

#Generate cases and controls

AAAff=0; ABAff=0; BBAff=0;

AAUna=0; ABUna=0; BBUna=0;

for (i in 1:ncases){

t=runif(1);

if(t<=one){AAAff=AAAff+1};

if ((one<t) & (t <=two)){ABAff=ABAff+1};

if (two<t){BBAff=BBAff+1};

}

for (i in 1:ncontrols){

t=runif(1);

if(t<=onea){AAUna=AAUna+1};

if ((onea<t) & (t <=twoa)){ABUna=ABUna+1};

if (twoa<t){BBUna=BBUna+1};

}

## P-val of genotype-disease association using logistic regression

y=array(c(AAAff,ABAff,BBAff,AAUna,ABUna,BBUna),dim=c(3,2));

gen=c("AA","AB","BB");

xframe=data.frame(y,gen);

regr=glm(y~gen,data=xframe,family=binomial);

# Wald test

design=model.matrix(regr);

beta.hat=regr$coef;

cov.beta=summary(regr)$cov.unscaled;

C=matrix(0,2,3);

C[1,2]=1;

C[2,3]=1;

var.C=C%*%(cov.beta%*%t(C));

chi2=t(C%*%beta.hat)%*%(solve(var.C)%*%(C%*%beta.hat));

pval=1-pchisq(chi2,2);

if (pval <=0.05) {sign=sign+1}

rep=rep+1;

if (rep>=iter){acaba=1};

}

POWER=100*sign/iter;

}

else {POWER=999};

FRR=round(FRR, digits=2);

PAF=round(PAF, digits=1);

POWER=round(POWER, digits=1);

out=c(PAF,FRR,POWER);

out

}
